# Distal Humerus Physeal Separation: Diagnostic Challenges and Implications

**DOI:** 10.7759/cureus.76248

**Published:** 2024-12-23

**Authors:** Wan Lye Cheong, Norazian Kamisan, Imma Isniza Ismail

**Affiliations:** 1 Orthopaedics and Traumatology, Universiti Putra Malaysia, Serdang, MYS

**Keywords:** distal humerus, epiphyseal separation, epiphysiolysis, physeal separation, transphyseal fracture

## Abstract

Distal humerus physeal separation is an uncommon and often misdiagnosed injury in infants and young children, frequently resulting in delayed treatment. We report three cases of distal humerus physeal separation that presented with different clinical scenarios with different management approaches. The first case describes a nine-month-old girl who was initially treated for presumed elbow cellulitis before presentation to our centre six weeks later. Conservative management, with further observation, noted remodelling of the distal humerus and full elbow range of motion (ROM) after 18 months. The second case involves a two-year-old girl with Kawasaki disease who underwent delayed closed reduction and percutaneous pinning after one week due to concurrent antiplatelet therapy. At four-month follow-up, there was cubitus varus deformity with slight limitation in elbow flexion. The third case is of a five-day-old male neonate with a right elbow deformity following an elective caesarean delivery at another hospital. Gentle manipulation was performed with a splint to improve alignment and immobilisation. The fracture united after five weeks. There was varus deformity but otherwise a full range of motion. These cases underscore the challenges in early diagnosis and management of distal humerus physeal separation in young children. Delayed treatment can lead to favourable outcomes, but follow-up is essential to observe for potential remodelling and residual deformity.

## Introduction

Distal humerus physeal separations, also referred to as transphyseal fractures of the distal humerus, involve the separation of the growth plate at the distal end of the humerus. They are exceptionally rare, with their incidence estimated at just 0.7% [1]. They commonly occur in children less than three years of age [2]. In neonates, the physis is smooth and transverse and represents the weakest part of the distal humerus, making it more prone to failure than bone, ligaments, or joint capsules. This structural weakness explains why physeal injuries are more common than elbow dislocations in this age group.

The condition was first described by Smith in 1850, with subsequent case reports and case series emerging sporadically over the years [3,4]. Mechanisms of injury include rotational shear forces or elbow hyperextension seen in difficult obstetric deliveries or non-accidental trauma, as well as falls on an outstretched arm. Very rarely, such injuries have also been observed in older athletic children following chronic repetitive stress [5]. It is also crucial to maintain a high index of suspicion for non-accidental trauma, as these injuries are associated with a 13-fold greater risk of child abuse compared to supracondylar humerus fractures [6].

A typical presentation includes elbow swelling, deformity, tenderness, muffled crepitus due to friction between cartilaginous instead of bony surfaces, and pseudoparalysis. Differential diagnoses should consider elbow joint dislocation, brachial plexus injury, septic arthritis, osteomyelitis, and metabolic bone diseases. Treatment options vary, ranging from immobilisation alone, closed reduction with or without percutaneous pinning, to open reduction with pinning. However, a consensus on the ideal treatment remains elusive due to the limited number of reported cases and the unclear long-term outcomes associated with these injuries [4,7].

Herein, we present a series of three patients who presented with different clinical scenarios with different management approaches and report the medium-term outcomes.

## Case presentation

Case 1

**Figure 1:**
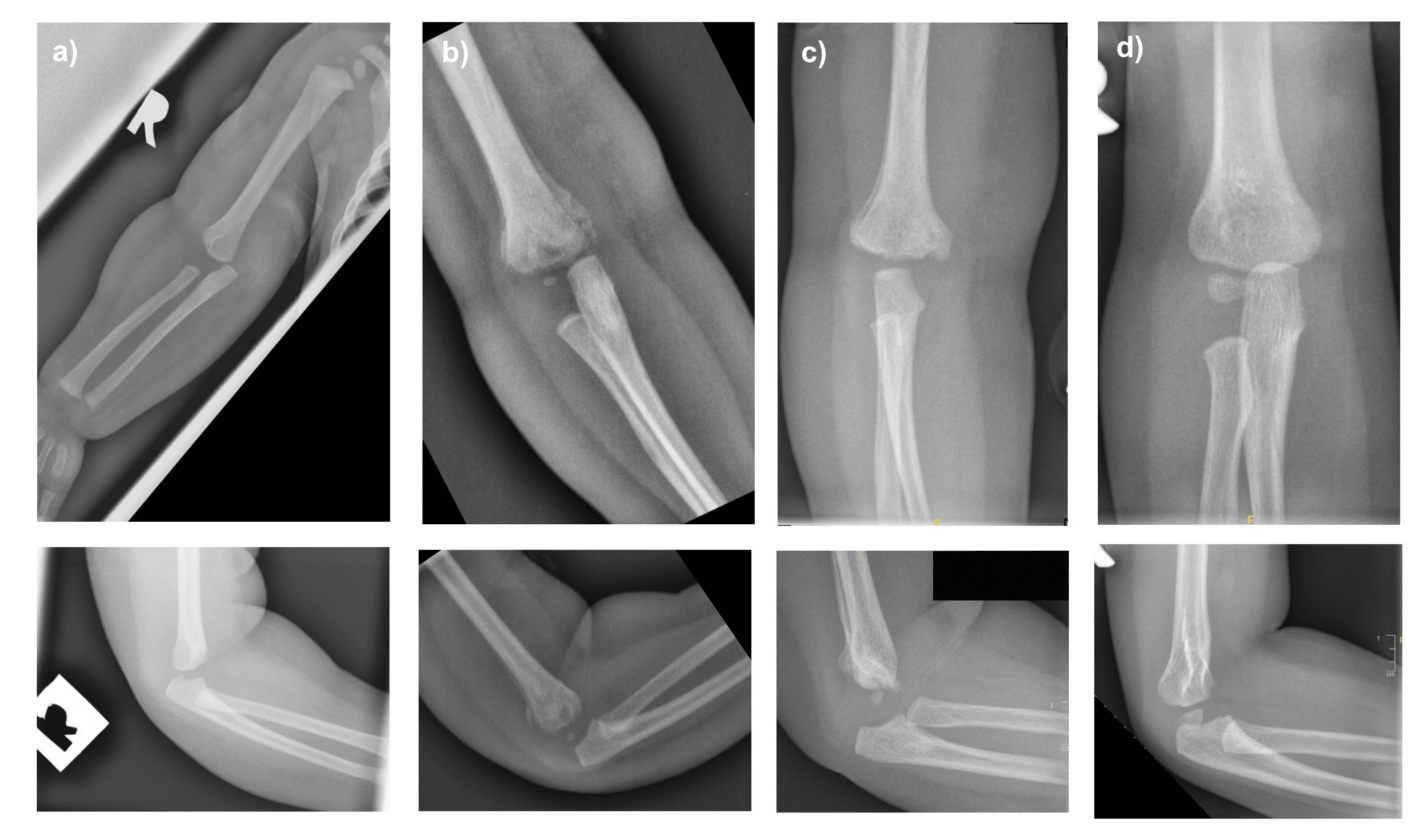
Serial elbow radiographs of case 1 Serial elbow radiographs at: (a) initial injury: showing posteromedial displacement of radius and ulna relative to distal humerus; (b) 3 weeks: showing callus formation and posterior metaphyseal spike; (c) 6 weeks: showing united fracture with residual posterior displacement of the forearm; (d) 18 months: showing a remodelled distal humerus.

A nine-month-old girl with a history of unilateral cleft lip and palate presented with right arm swelling for one day. She appeared fretful and resisted attempts to move her right upper limb. The swelling was first noticed after she returned home from a nursery. She had no fever or other symptoms suggestive of infection. She was otherwise active and feeding well. On examination, her right elbow was swollen with minimal bruise. The range of motion (ROM) of her right elbow was limited to 5° to 90° of flexion-extension. ROM of her right shoulder and wrist joints was full, and distal neurovascular status was intact. Her serum white blood cell count was 11 × 10^3^ cells/μl, and her C-reactive protein level was 8.4 mg/L. Ultrasonography of her right elbow reported features of cellulitis and myositis in the distal arm and proximal forearm, with no collection observed. She was initially treated at another hospital with intravenous antibiotics for presumed right elbow cellulitis and discharged after a week with a backslab. Upon referral to our centre six weeks after the initial presentation, she was found to have a fixed flexion deformity of 5° with limited elbow flexion up to 90°. Pronation and supination were preserved. Repeated radiographs showed callus formation around the distal humerus. After a review of her earlier radiographs, a retrospective diagnosis of distal humerus physeal separation was made (Figure 1). Her parents were informed regarding the diagnosis, expected recovery, and plan for observation as the child grows to monitor remodelling of the distal humerus and improvement in elbow ROM. At an 18-month follow-up, the distal humerus had united, and she regained a full ROM of her right elbow.

Case 2

**Figure 2:**
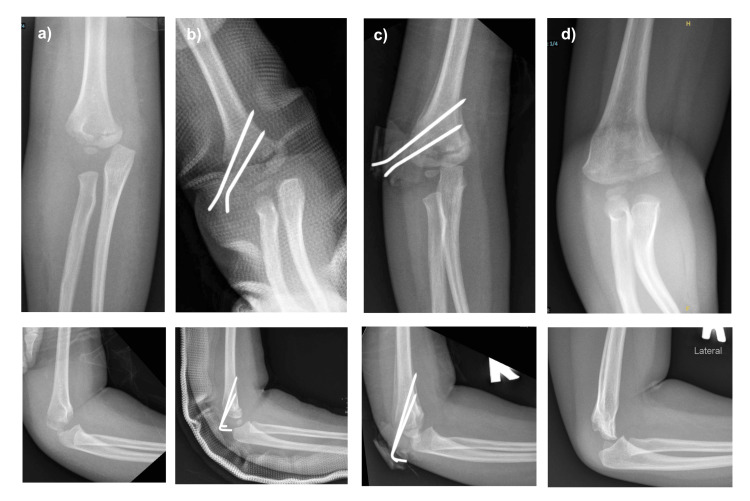
Serial elbow radiographs of case 2 Serial elbow radiographs at: (a) initial injury: showing posteromedial displacement of distal humerus epiphysis; (b) immediate post-operative radiograph following closed reduction and percutaneous pinning; (c) 3 weeks post-surgery: showing callus formation; (d) 4 months: showing a healed fracture with residual varus deformity on the coronal plane.

A two-year-old girl with underlying Kawasaki disease presented with right elbow pain following a fall from bed. On examination, there was bruising over her right elbow and limited ROM due to pain. The distal neurovascular examination was intact. A plain radiograph of her right elbow showed physeal separation of the distal humerus. Surgery was performed one week later as she was on double antiplatelet therapy, which needed to be discontinued prior to surgery after consultation with paediatric cardiology. An elbow arthrogram, closed manipulative reduction, and Kirschner wire fixation of the distal humerus were performed. Post-operative recovery was uneventful, and she was discharged home the next day with an above-elbow backslab. Follow-up radiographs at three weeks noted callus formation. The Kirschner wire and backslab were removed. The fracture united after six weeks (Figure 2). At four months post-surgery, there was a cubital varus deformity with elbow ROM of 0° to 110°. Follow-up was continued to observe for remodelling of the distal humerus.

Case 3

**Figure 3:**
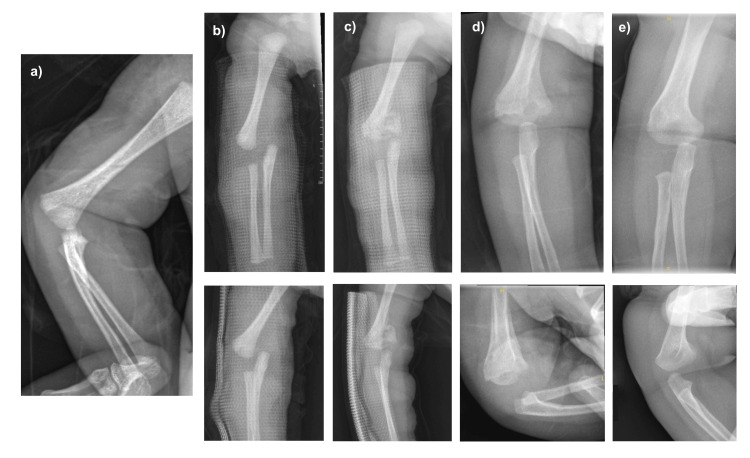
Serial elbow radiographs of case 3 Serial elbow radiographs at: (a) initial injury: showing medial displacement of the forearm axis compared to distal humerus and the oblique view presents a diagnostic difficulty; (b) day 5: showing medial displacement of the forearm axis in relation to the distal humerus; (c) 3 weeks: showing callus formation; (d) 5 weeks: showing a united fracture; (e) 4 months: showing remodelling of the distal humerus.

**Figure 4:**
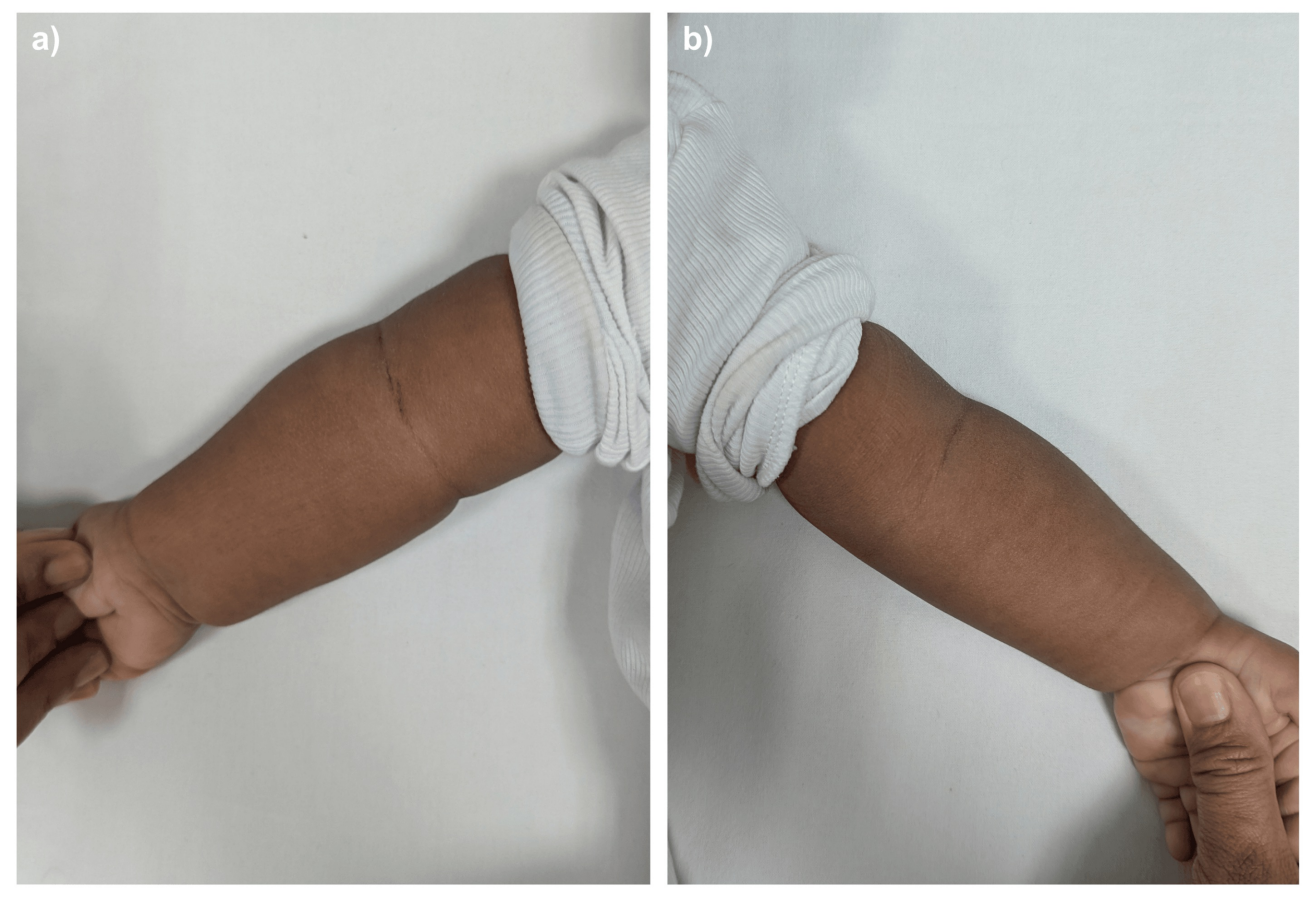
Clinical photo of (a) right elbow and (b) left elbow There is mild cubitus varus deformity of the right elbow compared to the contralateral elbow.

A five-day-old male neonate was referred from another centre following a birth injury during an elective caesarean section. After delivery, there was a deformity of his right upper limb with an incomplete Moro reflex. His birth weight was 3.08 kg. He was otherwise healthy. Upon our review, there was mild swelling over the right elbow. Hand movement and palmar grasp were good. Distal circulation was intact. Radiographs revealed a medially displaced ulna and radius in relation to the distal humerus. Gentle manipulation was performed to improve alignment, and an above-elbow backslab was applied. Two weeks later, repeat radiographs showed abundant callus formation. The backslab was removed. Five weeks after the injury, the fracture had united (Figure 3). After four months, he exhibited a mild cubitus varus deformity but retained full ROM (Figure 4). Follow-up was continued to observe for remodelling of the deformity.

## Discussion

Distal humerus physeal separations commonly occur in children of a younger age group whose distal humerus is still unossified. Due to this, the diagnosis can be difficult and often missed. In addition, a swollen elbow can obscure the anatomical landmarks distinguishing physeal separation from other diagnoses, such as elbow dislocation. In our first case, initial treatment for presumed cellulitis led to delayed recognition of physeal separation until callus formation and deformity were evident.

Nevertheless, there are tell-tale radiographic signs of a physeal separation of the distal humerus, which the clinician must look for. An important detail to remember is the onset of ossification of the capitellum, which is between three to nine months old [8]. When the capitellar ossification centre is visible, posteromedial displacement of the radius and ulna relative to the distal humerus, while maintaining normal radiocapitellar alignment, indicates physeal separation. However, an oblique view may mask the displacement [9]. The Thurston-Holland, or corner sign, which is a flake of bone accompanying the physeal separation, may also be seen [8]. This can be falsely attributed to a lateral condyle fracture, but again, careful evaluation will show a normal radiocapitellar alignment. In the absence of the capitellar ossification centre, a useful finding is a decreased gap between the radial metaphysis and anterior humeral line on a lateral radiographic view in comparison to the contralateral elbow [10]. Additionally, with late presentation, periosteal new bone formation may be seen after seven to ten days [8,11]. In contrast, an elbow dislocation, which is often the wrong diagnosis, usually presents with posterolateral displacement [11]. Here, the humerus and forearm bony axes are displaced, while the capitellum remains in line with the distal humerus.

There are other imaging modalities that can aid in diagnosis. Ultrasonography is safe, non-ionising, non-invasive, and effective in visualising cartilaginous epiphyseal translation [9]. However, it requires a skilled operator and may produce discomfort and pain. An arthrogram, which highlights unossified cartilaginous structures, is useful intra-operatively to assess the quality of closed reduction and determine the need for an open reduction [12]. Its disadvantages are the invasive nature of the procedure, contrast extravasation due to capsular tear, and risk of infection. Magnetic resonance imaging (MRI) can provide a detailed multiplanar view of the bone, cartilage, and soft tissue structures. However, it may not be readily available, require time for preparation, and children may require sedation, ultimately leading to a delay in diagnosis and treatment. In our practice, plain radiographs sufficed for diagnosis, while an arthrogram was used to assist with closed reduction intra-operatively. In centres without a paediatric orthopaedic unit, the aforementioned adjuncts may greatly increase the rate of correct diagnosis.

The goal of treatment is to maintain acceptable alignment until fracture healing in two to three weeks while avoiding future complications. The commonest complication is cubitus varus deformity, which is usually non-progressive. Other complications include avascular necrosis of the medial humeral condyle, growth plate injury, reduced ROM, myositis ossificans, and non-union. Treatment methods can be grossly divided according to age, amount of displacement, and timing of presentation. Neonates demonstrate excellent outcomes in terms of carrying angle and ROM due to their remodelling capacity. Thus, Jacobsen et al. suggested that manipulation or operative treatment is not necessary in neonates. In children above six months old, however, 35% developed varus deformity [7]. This may suggest that an intervention to improve the alignment should at least be attempted in older children. On the other hand, Oh et al. noted that although cubitus varus was a common complication in 12 patients, it was not related to their age or methods of treatment [13]. This is corroborated by Zhou et al., who also did not find a significant association between age and type of reduction with the functional outcome [12].

When the fracture is minimally displaced, immobilisation with a cast or splint alone may be adequate. However, in patients with severe displacement of more than 35%, pin fixation after reduction may provide better results [14]. If the reduction cannot be achieved via the closed method, an open reduction may be necessary, although there is a risk of devascularising the physeal fragment.

In our first case, the child presented late after six weeks with callus formation on radiograph. Although there was initial flexion deformity, it resolved after 18 months. This highlights the remodelling potential in younger children. In the second case of our series, treatment was delayed by a week due to her comorbidity. Previous authors have advised against late manipulation beyond four to seven days as the epiphysis is now less mobile, leading to an increased risk of avascular necrosis and physeal injury. They advocated treating the resulting deformity at a later stage with corrective osteotomy rather than risk avascular necrosis or premature physeal arrest. However, a recent series by Wu et al. noted acceptable results for 12 patients with delayed presentation (7-20 days) treated with closed reduction and percutaneous pinning. Only one child who was six years old and presented after 30 days had a poor outcome, with a 40° loss of ROM and a 20° carrying angle difference [15]. We performed gentle manipulation followed by splint immobilisation in our third case, as we believe there is great remodelling potential in neonates. Furthermore, Cha et al. proposed minimal anatomical reduction in children less than two years old to preserve the vascular supply to the medial condyle. This is in contrast to toddlers, who require a precise anatomical reduction to prevent malunion, which is a common cause of cubitus varus deformity rather than physeal growth disturbance [16].

Given the rarity of this injury, establishing a definitive treatment guideline remains a challenge. However, outcomes generally appear favourable when management strategies are tailored to the child’s age, degree of displacement, and timing of presentation [4,7,14]. Further research and case studies are needed to gather more data and refine treatment approaches.

## Conclusions

Physeal separation of the distal humerus is a rare injury often prone to a missed or incorrect diagnosis. Careful evaluation of radiographs is essential for a correct diagnosis. Adjuncts such as ultrasonography and magnetic resonance imaging are helpful tools when the diagnosis is unclear. Prompt and accurate diagnosis is required to avoid unnecessary or inadequate surgical intervention while allaying parental anxiety. Functional outcome is good despite the relatively high incidence of cubitus varus deformity.
